# A funny thing happened on the way to the journal: a commentary on Foucault's ethics and Stuart Murray's "Care of the self"

**DOI:** 10.1186/1747-5341-3-2

**Published:** 2008-01-14

**Authors:** Madeleine J Murtagh

**Affiliations:** 1 Institute of Health and Society, University of Newcastle, Newcastle upon Tyne, UK

## Abstract

Stuart Murray's 'Care and the self: biotechnology, reproduction, and the good life' utilizes Foucault's "care of the self" to examine health domains in its title. The present author discusses three important articulations of concern with the Foucauldian concepts of care of the self that are absent in the work of Murray and others: first, the voluntarism and individualism inherent in ideas about care of the self; second, the absence of the interactional and relational; and, third, the perpetuation of the interpretation of Foucault's concept of governmentality, 'the conduct of conduct', as primarily coercive.

## Commentary

"Something familiar, something peculiar, something for everyone" is the promise of the opening number in Stephen Sondheim's Broadway musical "A Funny Thing Happened on the Way to the Forum." And so it is with the recent paper in this journal by Stuart Murray, 'Care and the self: biotechnology, reproduction, and the good life' [1]. We find here both the familiar and the peculiar in the presentation of Foucault's 'care of the self'. But is there something for everyone?

Stuart Murray's paper is to be welcomed for his discussion of the potential for a Foucauldian inspired ethics following ideas about 'care of the self'. There is little doubt that a rethinking and reworking of the enlightenment subject in bioethics is necessary. Indeed Murray is not alone in presenting a critique of autonomy (see Frank [2], for a review of critiques of autonomy that coalesce in the late 1990's) and suggesting a turn towards a Foucauldian ethics (cf. McNay [3] and the special issue of Journal of Medical Humanities [4]). Rather than establish the argument for such an ethics, this commentator would have been heartened by a progression of these ideas and an exploration of how such an ethics could be enacted. Is it possible, or desirable, that these ethics replace, enhance, or complement contemporary bioethics?

Murray's manuscript contains clues to a direction for a Foucauldian ethics. The explication of the interaction of Socrates with his student is an enticing injunction to come to know one's self, to know "the relationship between the self and itself, which includes the relation between the self and others whose love and wisdom helps to bring that self into a caring proximity with itself" [1]. Importantly, as emphasized by Foucault, such knowledge of the self is not without interaction beyond the self [5,6]. However, in the process of presenting an argument for such a turn in biomedical ethics, the objects of criticism are insufficiently drawn by Murray and we are left with the portrait of a uni-dimensional world in which medicine and medics are bad, the public dupes, and Foucault the solution. This presentation leaves us in an uncomfortably disempowered position as we are likely to be one or the other of these subjects. Moreover, if critiques of modernism teach us anything it is that universalising constructs are not conceptually or practically workable. Further, such a presentation logically precludes the engagement of a 'malign' medicine and a 'passive' public in care of the self. We are not therefore encouraged to engage with three important concerns with the Foucauldian concepts in play in Murray's paper: particularly governmentality and care of the self. These concerns include, the voluntarism and individualism inherent in ideas about care of the self, the absence of the interactional and relational and, the perpetuation of the interpretation of Foucault's concept of governmentality, 'the conduct of conduct', as primarily coercive.

First, the concept of 'care of the self' used by Murray and others is voluntaristic, one that enjoins the individual to make of themselves a project and a place for reflection, to engage in an 'aesthetics of the self'. This involves conscious and critical reflection on the social world and engagement on one's own terms rather than those laid down in advance as rules of conduct: an upending of those rules and a creation, through active and thoughtful reflection, of a new mode of conduct. A practical and ethical anarchy, if you like. The outcome of such an ethics is the opportunity for individuals to change their relationship to the symbolic order; it is a way out of the constraints of socially constructed conceptions of, for example, femininity and masculinity, and of "the other." It does not, however, account for the material constraints and the social circumstances that allow different opportunities for individuals. In so doing it potentially reproduces existing social inequalities; as, it must be noted, do many approaches to addressing inequalities that favour the educated, articulate and materially fortunate of the populace. Importantly, the 'care of the self' articulated through the self promotes an ethics that in its individualism may fail to account for the interaction of the individual in society; for their responsibility within that society; and for the effects of their lives upon that society. I have elsewhere argued, after McNay [3] and Abrams [7], for a 'care of the self' that is both generative and transformative. Generative as an ethics in that its attentiveness to the symbolic and material dimensions of the social world enables a dynamic theory of agency which is capable of explaining how individuals are able to act in creative and unanticipated ways upon complex social relations. Transformative in that it is an ethics that recognises group-based oppression and produces a form of agency that targets institutions and their practices and may incorporate individual or collective action to disrupt social or cultural practices.

Second, the 'care of the self' is commonly conceived of in the absence of the interactional and relational. In his exposition on the 'care of the self' through the device of Socrates' conversation with Alchibiades, Murray proposes an ethics of the 'self' that must go beyond the relationship of self to self. We are nonetheless left disappointed, as the promise of a relational ethics following Foucault is not fully conceived. So what would such a relational ethics look like? It might, for example, look to Sennet's injunction to turn outward, to engage in the mistaken recognition of the other as self and in so doing develop mutuality and reciprocity as part of an ethics of respect [8]. We might in a relational ethics of the self follow Bergum and Dossetor in describing relational ethics (in this case in healthcare) as a practice grounded in relationships: in the specific case of healthcare, the relationships between care providers and patients, between patients and their families, between theorists and practitioners, and even between the advocates of competing positions and systems [9]. Bergum and Dossetor argue it is the relationship itself that supports and informs ethical reflection and decision making. Much like Socrates of Alchibiades, being attentive and responsive is the basis of this approach. Being sensitive to different life circumstances and perspectives of individuals, families and communities is essential. The core elements of relational ethics are meaningful interaction, mutual respect, uncertainty and vulnerability and an interdependent environment. In Sennet's terms, "the problem we face is how the strong can practice respect towards those destined to remain weak" (p 119).

Third, the predominant understanding of 'the conduct of conduct' in governmentality studies is as a technology of power that is coercive. It is upon this understanding that Murray draws as he states "While we are thoroughly beholden to the terms of modern medicine, and while the self is interpellated as a subject of medical authority, medicine continues to sell itself as "self"-empowering." [1]. Such an interpretation of Foucault's governmentality is common and typically follows Nicolas Rose's conception of "forms of freedom" in which the individual is "bound to a regime of subjectification in which subjects are not merely 'free to choose' but obliged to be free, to understand and enact their lives in terms of choice under conditions that systematically limit the capacity of so many to shape their own destiny" [10] (p17). This is a powerful argument for unseating unexpected and counterintuitive technologies of power (cf, Murtagh and Hepworth [11], amongst others). It is not, however, sufficient, as I have previously done myself, to simply regard governmentality as a constraint on freedom and for the apparent coercion of the 'conduct of conduct' to be understood as uniformly negative. Governmentality is a constraint on freedom only if we insist on understanding freedom in terms of the individual. Mechanisms of power and power relations are necessarily local, specific and, if we are to read them through Foucault's later work, embedded within the mechanisms of the security of populations [10]. Mechanisms of power have both positive and negative effects; they constrain, maintain and produce certain ways of being and living. We need to develop a more nuanced understandings of the practices of governmentality; understandings which are themselves not reliant upon individualistic notions of ethics but rather take account of individuals in interaction, that is, a governmentality of and in populations that has both negative and positive aspirations and effects. Murray's injunction to develop a Foucauldian ethics holds promise but is limited by its attachment to the negative paradigm of governmentality.

For Foucault, "Freedom is the ontological condition of ethics. But ethics is the considered form that freedom takes when it is informed by reflection" [6](p. 284). This considered form need not be voluntaristic and individualistic; it need not exclude the relational or interactional; it is not, as a form of self governance necessarily coercive but then neither is it necessarily beneficial or beneficent. My challenge then to Stuart Murray and others (I have to say, myself included) is to take the argument further; leave us not with the anticlimax of 'what now?' Give us "something for everyone"; just not the universal subject.
